# Molecular heterogeneity of glioblastomas: does location matter?

**DOI:** 10.18632/oncotarget.6433

**Published:** 2015-11-30

**Authors:** Emilie Denicolaï, Emeline Tabouret, Carole Colin, Philippe Metellus, Isabelle Nanni, Celine Boucard, Aurélie Tchoghandjian, David Meyronet, Nathalie Baeza-Kallee, Olivier Chinot, Dominique Figarella-Branger

**Affiliations:** ^1^ Aix-Marseille University, CRO2 UMR 911, Inserm UMR_S 911, Marseille, 13385, France; ^2^ AP-HM, Timone Hospital, Department of Neuro-Oncology, Marseille, 13385, France; ^3^ AP-HM, Timone Hospital, Department of Neuro-Surgery, Marseille, 13385, France; ^4^ AP-HM, North Hospital, Transfer Laboratory, Marseille, 13915, France; ^5^ Hospices Civils de Lyon, Centre de Pathologie et de Neuropathologie Est, Lyon, 69677, France; ^6^ AP-HM, Timone Hospital, Department of Anatomopathology, Marseille, 13385, France

**Keywords:** tumor location, glioblastoma, mesenchymal profile, proneural profile

## Abstract

Glioblastomas in adults are highly heterogeneous tumors that can develop throughout the brain. To date no predictive-location marker has been identified. We previously derived two glioblastoma cell lines from cortical and periventricular locations and demonstrated distinct transcriptomic profiles. Based on these preliminary results, the aim of this study was to correlate glioblastoma locations with the expression of ten selected genes (*VEGFC*, *FLT4*, *MET*, *HGF*, *CHI3L1*, *PROM1*, *NOTCH1*, *DLL3*, *PDGFRA*, *BCAN*). Fifty nine patients with newly diagnosed glioblastomas were retrospectively included. Tumors were classified into cortical and periventricular locations, which were subsequently segregated according to cerebral lobes involved: cortical fronto-parietal (C-FP), cortical temporal (C-T), periventricular fronto-parietal (PV-FP), periventricular temporal (PV-T), and periventricular occipital (PV-O). Gene expression levels were determined using RT-qPCR. Compared to cortical glioblastomas, periventricular glioblastomas were characterized by a higher expression of two mesenchymal genes, *VEGFC* (*p* = 0.001) and *HGF* (*p* = 0.001). Among cortical locations, gene expressions were homogeneous. In contrast, periventricular locations exhibited distinct expression profiles. PV-T tumors were associated with higher expression of two proneural and cancer stem cell genes, *NOTCH1* (*p* = 0.028) and *PROM1* (*p* = 0.033) while PV-FP tumors were characterized by high expression of a mesenchymal gene, *CHI3L1* (*p* = 0.006). Protein expression of NOTCH1 was correlated with RNA expression levels. PV-O glioblastomas were associated with lower expression of *VEGFC* (*p* = 0.032) than other periventricular locations, whereas *MET* overexpression remained exceptional. These data suggest a differential gene expression profile according to initial glioblastoma location.

## INTRODUCTION

Glioblastomas are the most common and aggressive primary brain tumors in adults. Despite increasing numbers of new innovative therapeutic approaches, the recurrence remains inevitable. For the moment, first line treatment includes radiotherapy plus concomitant and adjuvant temozolomide. This treatment regimen has been shown to increase median overall survival (OS) from 12.1 to 14.6 months [1]. Recently, the addition of bevacizumab to standard glioblastoma treatment was evaluated in two large phase III trials, demonstrating improvement of progression free survival (PFS) but no OS benefit [2, 3]. To date, these results did not lead to modification of standard of care.

Understanding of molecular and cellular characteristics involved in gliomagenesis process remains a corner stone of therapeutic developments. Glioblastomas were characterized by a major molecular heterogeneity based on genetic diversity, cell origin variability and strong interactions with local microenvironment [4, 5]. Molecular characterizations of these tumors have led to propose molecular classification of glioblastomas into three subgroups: proneural, mesenchymal, and proliferative [6]. In addition, The Cancer Genome Atlas (TCGA) classified glioblastomas on the basis of *PDGFRA*, *IDH1*, *EGFR*, and *NF1* abnormalities in classical, mesenchymal, proneural, and neural subtypes, and these four subgroups seemed to present distinct outcomes [7–9]. More recently, another classification related to methylation profile was also reported [10]. Furthermore, glioblastomas display high cellular heterogeneity based on the hypothesis of cancer stem cells (CSC) and precursor cell populations, leading to glioblastoma initiation, treatment resistance, tumor recurrence and invasiveness ability [11, 12]. Various CSC surface markers have been described in glioblastomas, including the most frequently represented CD133 (PROM1), A2B5, and CD15 (stage-specific embryonic antigen 1) [13–16]. Although, some clinical studies showed that only the glycoprotein CD133 had a prognostic value [17]. CSC subtypes with distinct gene expression profiles and growth patterns have also been reported in glioblastomas [18, 19]. In a previous study, we established two glioblastoma stem-like cell lines from periventricular and cortical tumor locations, and we observed specific *in vitro* and *in vivo* behaviors [20]. In particular, transcriptomic analyses showed a mesenchymal profile for periventricular glioblastoma stem-like cell line, with overexpression of *VEGFC* and *CHI3L1* and a proneural profile for cortical glioblastoma stem-like cell line with overexpression of *PROM1*, *DLL3*, and *BCAN*.

Based on these preliminary results and the published molecular classifications which highlighted two main genetic subtypes (mesenchymal and proneural) [6–8], the aim of the present study was to evaluate the expression of five mesenchymal genes (*VEGFC*, *FLT4*, *MET*, *HGF*, *CHI3L1*), and five proneural genes (*PROM1*, *NOTCH1*, *DLL3*, *PDGFRA*, *BCAN*) in a series of 59 newly diagnosed glioblastomas. We postulate that, according to their location, glioblastomas display differential gene expression profiles.

## RESULTS

### Patient characteristics

Fifty nine patients were included in this study, with a median age at diagnosis of 61.3 years (range, 20.5–78.2). At diagnosis, 46 (78%) patients presented conserved general status (KPS ≥ 70). Gross total surgical resections were performed in 40 patients (67.8%) and diagnostic biopsies were taken from the remaining 19 patients (32.2%). Radio-chemotherapy was used as the first line treatment for 72.9% of the patients. All tumors were IDH1 R132H negative. *MGMT* promoter methylation status was known for 51/59 patients: the *MGMT* promoter was methylated in 16/51 tumor samples (31%). *EGFR* amplification status was known for all patients: *EGFR* was amplified in 27/59 (46%) tumor samples. Regarding the tumor location, 24 (40.7%) patients belonged to cortical tumor group and 35 (59.3%) to periventricular tumor group. Among cortical tumors, 19 (32.2%) were fronto-parietal and 5 (8.5%) temporal, whereas in periventricular subgroup, 12 (20.3%) tumors were fronto-parietal, 12 (20.3%) temporal and 11 (18.7%) occipital (Table 1).

**Table 1:** Patients characteristics (*n* = 59)

Characteristics	*n* = 59	(%)
**Median age**, years (range)	61.3 (20.5–78.2)	
**Gender**		
Male / Female	36 / 22	(62 / 38)
**KPS score**		
≥ 70	46	(78)
< 70	13	(22)
**Type of surgery**		
Gross total resection	40	(67.8)
Other (Partial exerese, biopsy)	19	(32.2)
**First line treatment**		
Radio-chemotherapy	43	(72.9)
Radiotherapy alone	4	(6.8)
Alkylating agents	11	(18.6)
**Molecular data**		
MGMT methylated / unmethylated	16 / 35	(31 / 69)
EGFR amplification	27	(46)
**Location**		
Cortical	24	(40.7)
Cortical fronto-parietal	19	(32.2)
Cortical temporal	5	(8.5)
Periventricular	35	(59.3)
Periventricular fronto-parietal	12	(20.3)
Periventricular temporal	12	(20.3)
Periventricular occipital	11	(18.7)

Abbreviations: O(6)-Methylguanine-DNA methyltransferase (MGMT) and Epidermal growth factor receptor (EGFR).

### Molecular marker expression and tumor location

#### Cortical versus periventricular location

**Figure 1:**
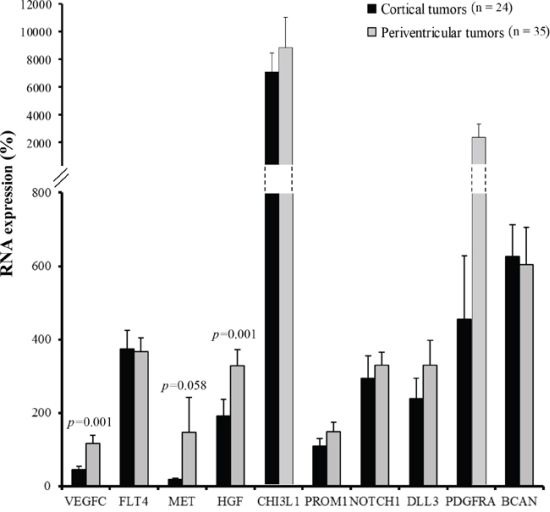
Cortical tumors (dark grey, n = 24) *versus* periventricular tumors (light grey, n = 35) RNA expression profile *VEGFC*, *FLT4*, *MET*, *HGF*, *CHI3L1*, *PROM1*, *NOTCH1*, *DLL3*, *PDGFRA*, and *BCAN* RNA expression were quantified by RT-qPCR. Expression levels were expressed as percentages of expression compared to normal adult human brain samples. Bars indicate the Standard Error of the Mean (SEM). Correlations between tumor locations and RNA expression profiles were analyzed using non-parametric Mann-Whitney tests.

To identify putative differential expression patterns according to glioblastoma location, *VEGFC*, *FLT4*, *MET*, *HGF*, *CHI3L1*, *PROM1*, *NOTCH1*, *DLL3*, *PDGFRA*, and *BCAN* RNA expression levels were compared between cortical (*n* = 24) and periventricular tumor samples (*n* = 35). *VEGFC* and *HGF* markers displayed a significant differential expression between cortical and periventricular locations, (*p* = 0.001 and *p* = 0.001, respectively), and *MET* expression tended to differ between locations (*p* = 0.058) (Figure 1).

#### Up to five tumor locations identification

**Figure 2:**
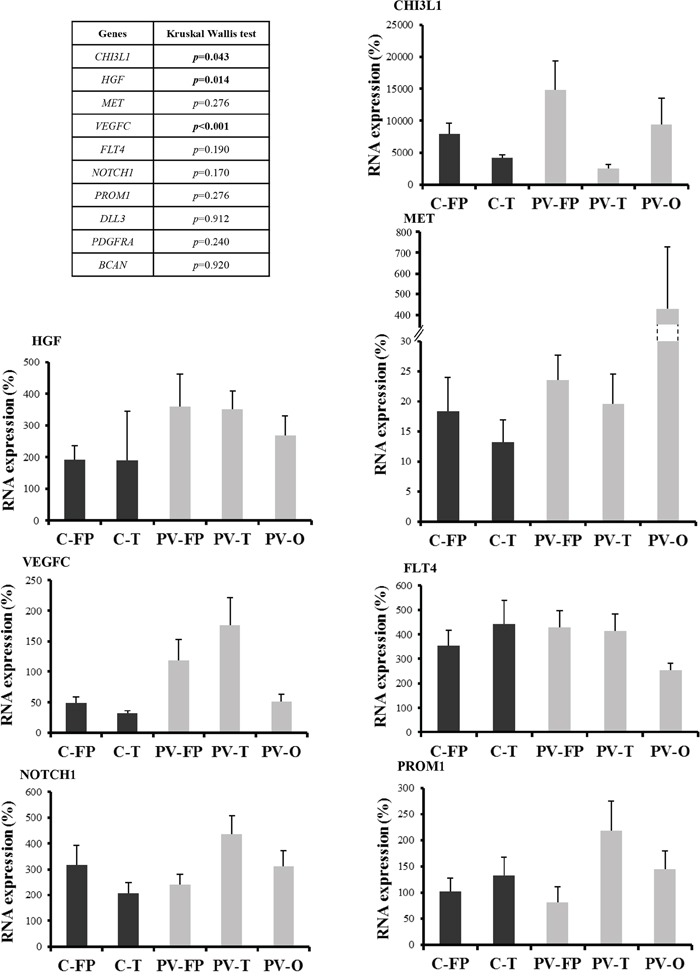
RNA expression profiles according to the five tumor locations RNA expression was quantified by RT-qPCR. Expression levels were expressed as percentages of expression compared to adult human brain samples. Correlations between tumor locations and RNA expression profiles were analyzed using non-parametric Kruskal-Wallis test. Only RNA expression levels significantly different between the five glioblastoma locations were showed. *P*-values are given in the left panel. Abbreviations: Cortical fronto-parietal (C-FP), Cortical temporal (C-T), Periventricular fronto-parietal (PV-FP), Periventricular temporal (PV-T), Periventricular occipital (PV-O). Bars indicate the SEM.

**Figure 3:**
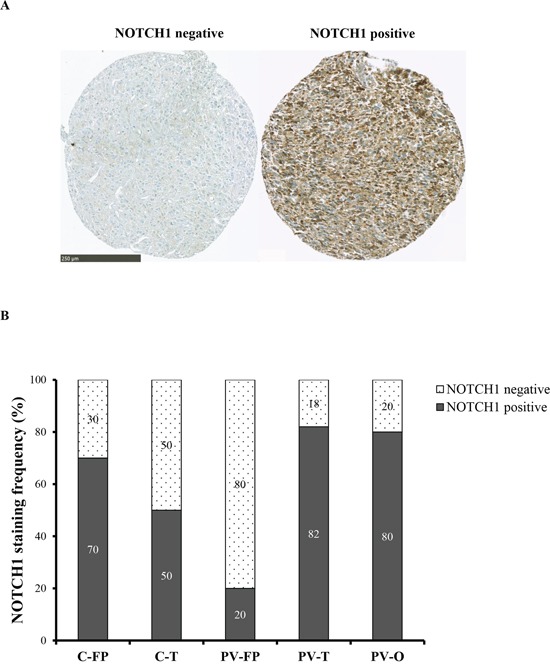
NOTCH1 expression according to the five tumor locations **A.** NOTCH1 immunostaining was performed on TMA. Left: negative expression. Right: positive expression is seen mainly in cytoplasm and rarely in nucleus of the positive cells. Scale bar: 250 μm. **B.** NOTCH1 staining frequency (%) is represented in each five tumor locations. NOTCH1 positive tumors are predominant in PV-T location and NOTCH1 negative tumors in PV-FP location.

**Figure 4:**
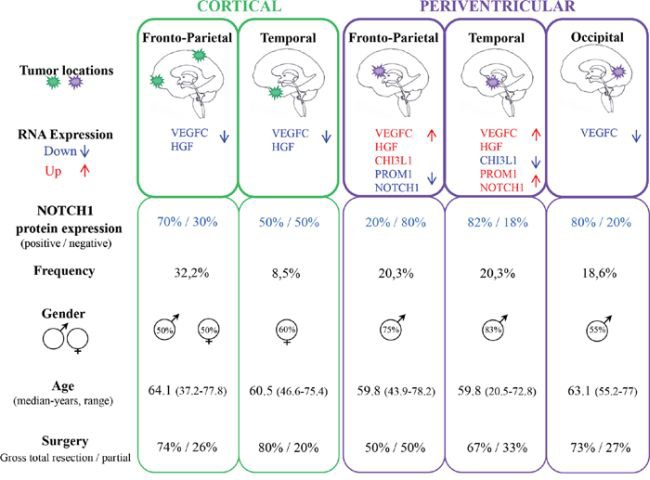
Main clinical and molecular features of the five tumor locations

Tumor locations were analyzed according to cerebral lobe involvement and ventricular anatomy. Cortical tumors were dichotomized into fronto-parietal (C-FP) and temporal (C-T) locations, and periventricular tumors were divided into fronto-parietal (PV-FP), temporal (PV-T), and occipital (PV-O) locations. *VEGFC* (*p* < 0.001), *HGF* (*p* = 0.014), and *CHI3L1* (*p* = 0.043) RNA expression levels were significantly different between the five glioblastoma locations (Figure 2 and Supplementary Table S1). Moreover, we specifically identified significant differences in the expression of *VEGFC*, *FLT4*, *PROM1*, *NOTCH1*, and *CHI3L1* between distinct subgroups of periventricular tumors (Supplementary Table S2). In contrast, no difference was observed between the two cortical locations. Finally, *DLL3*, *PDGFRA*, and *BCAN* were heterogeneously distributed within the five subgroups (Figure 2 and Supplementary Table S1). Accordingly, periventricular locations exhibited distinct expression profiles. In particular, *VEGFC* expression was significantly higher in PV-T and PV-FP tumors than in PV-O tumors (p = 0.004 and p = 0.032, respectively). In contrast, mean *MET* expression appeared lower in PV-FP (23 ± 4.1%), and PV-T (19 ± 4.9%) locations than in the PV-O (419 ± 292.9%) location (*p* = 0.276). Mean *MET* expression in the PV-O subgroup reflected two tumor samples with very high *MET* expression (1259% and 3126%). Moreover, PV-T tumors were associated with higher expression of two proneural and cancer stem cell genes, *PROM1* (*p* = 0.033), and *NOTCH1* (*p* = 0.028) while PV-FP tumors were characterized by a higher expression of *CHI3L1* (*p* = 0.006) (Figure 2 and Supplementary Table S1). Interestingly, protein expression analysis of NOTCH1 showed a significant difference between the five glioblastoma locations (*p* = 0.037) and was associated with RNA expression levels (*p* = 0.004). The NOTCH1 positive staining was mostly found in cytoplasm of positive cells (Figure 3A). NOTCH1 positive staining was higher in PV-T location (82%) and lower in PV-FP location (20%) (Figure 3B). Clinical characteristics and gene expression levels of the five main tumor locations are summarized in Figure 4.

### Correlation between marker expressions

By the Spearman correlation test, *VEGFC* RNA expression was correlated with that of *HGF* (*p* < 0.001), while *NOTCH1* expression was correlated with that of *PROM1* (*p* < 0.001) and inversely with that of *CHI3L1* (*p* = 0.003). Other correlations related to marker expression are listed in Supplementary Table S3.

## DISCUSSION

In this study, we correlated glioblastoma locations with the expression of five mesenchymal genes (*VEGFC* and its receptor *FLT4*, *HGF* and its receptor *MET*, and *CHI3L1*) and five proneural genes (*PROM1*, *NOTCH1*, *DLL3*, *PDGFRA*, and *BCAN*). Initially, glioblastomas were classified as cortical or periventricular lesions according to previous reports [20, 21], and they were then separated into five subgroups according to cerebral lobes: two cortical locations (C-T and C-FP) and three periventricular locations (PV-FP, PV-T, and PV-O).

Among genes, *DLL3*, *PDGFRA* and *BCAN* were heterogeneously distributed across subgroups. Therefore, we focused our study on the seven remaining markers. Expression of the two mesenchymal markers, *VEGFC* and *HGF*, was significantly higher in periventricular glioblastomas than in cortical glioblastomas. We showed in our previous study that a mesenchymal signature was recorded among periventricular glioblastomas, whereas cortical glioblastomas displayed a classical or proneural molecular profile [20]. In a recent study, MRI data were correlated to molecular profiles of patients classified according to the TCGA subgroups. The extent of contrast enhancement was significantly associated with proneural and mesenchymal molecular subtypes. Specifically, the mesenchymal subtype showed more diffuse contrast enhancement than the proneural subtype [22]. Although these authors did not correlate glioblastoma locations (cortical *versus* periventricular) with genetic signatures, these data clearly suggest distinct growth properties between proneural and mesenchymal glioblastomas.

The *VEGFC* gene encodes one of the most important proteins involved in the activation of tumor angiogenesis and lymphangiogenesis [23], and its expression is strongly associated with migration and metastasis of many cancers [24]. Its main receptor, FLT4, was also strongly expressed in all glioblastomas of the present study, regardless of location. Previous descriptive MRI studies indicated that glioblastomas of subventricular zones (periventricular glioblastomas) were more likely to be multifocal at diagnosis, and recurred at greater distances from initial lesions than cortical glioblastomas [25]. Therefore, strong *VEGFC* expression in periventricular tumors might contribute to their high infiltrative properties and especially in PV-FP and PV-T tumors in contrast to cortical tumors with a low *VEGFC* expression. In addition, high *HGF* expression was observed in periventricular glioblastomas. Binding of HGF to its MET receptor triggers a series of intracellular signaling events leading to tumor cell proliferation, survival invasion and angiogenesis (for review see [26]). In our series, *MET* expression was not related to *HGF* expression and only two cases of the PV-O location exhibited highest mean *MET* value. In contrast to the four other locations, PV-T subtype expressed the proneural markers *PROM1* and *NOTCH1* at high levels. Moreover, *PROM1* (also known as *CD133*) is a well characterized CSC marker, although previous studies also report CSC properties in CD133^−^ cells [13]. Moreover, the NOTCH signaling pathway is known to maintain neural stem cells [27], and stimulate cell proliferation, cell differentiation, and tumorigenesis of glioma stem-like cells [28–30]. Therefore, high expression of these two markers suggests critical roles of CD133^+^ stem-like cells in the growth of this PV-T glioblastoma subtype. Theses results were supported by protein expression analysis of NOTCH1. Importantly, these PV-T tumors are close to the hippocampus, localized between the temporal lobe and the inferior horn of the lateral ventricle. Adult neurogenesis, initiated by neural stem cells, occurs in some specific brain areas such as the subgranular zone of the dentate gyrus of the hippocampus and the subventricular zone [31, 32]. Some recent studies revealed the presence of neural stem cells CD133^+^ in the adult murine hippocampus [33], and showed a potential relationship between NOTCH1 expression and neurogenesis in hippocampus [34, 35]. Interestingly, Han *et al*. [36] showed that adult hippocampal neural stem cells expressed VEGFC and its receptor FLT4, which represent specific regulators of neural stem cells activation and neurogenesis in mammals.

In summary, periventricular glioblastomas differed from cortical glioblastomas, and periventricular tumors were a heterogeneous group with distinct subgroups based on molecular characteristics. In particular, in contrast with PV-FP and PV-O subtype, PV-T glioblastomas were characterized by a high expression of the mesenchymal marker *VEGFC* and the proneural and CSC markers *NOTCH1* and *PROM1* that could be potential targets for specific therapies. Whether these data suggest that glioblastomas from PV-T location derived from specific Glioma stem-like cells this remains to be demonstrated. Limited numbers of cases studied and intratumoral heterogeneity [37] indicate the requirement of further studies to support present observations.

## MATERIALS AND METHODS

### Patient selection and clinical data (Figure 5)

All patients referred to our institution were retrospectively included if they met the following criteria: adult patient (≥18 years old) at the time of glioblastoma diagnosis, available pre-surgery MRI and frozen tumor sample stored in the Assistance Publique-Hôpitaux de Marseille (AP-HM) tumor bank (authorization number AC-2013–1786). Patients with only pre-surgery CT scans or post-surgery MRI were not included. In order to analyze the correlation between tumor location and molecular profile, we excluded patients with multifocal lesions or with huge tumors with both cortical and ventricular involvement precluding initial location determination. Subsequently, gender, Karnofsky performance status (KPS) at diagnosis, extent of surgical resection, and adjuvant treatments were recorded for all included patients.

**Figure 5:**
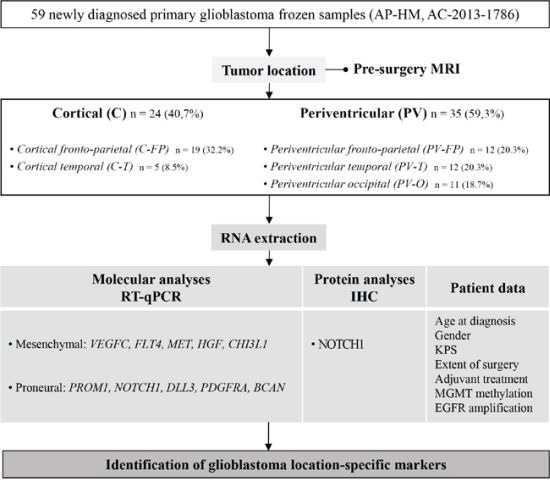
Study flow chart and objectives Abbreviations: Immunohistochemistry (IHC), Karnofsky performance status (KPS), O(6)-Methylguanine-DNA methyltransferase (MGMT) and Epidermal growth factor receptor (EGFR).

### Tumor samples, immunohistochemical and molecular data

Tumor tissue samples were obtained at the time of initial surgical resection and after written consent, according to a protocol approved by the local institutional review board and ethics committee. Tumor samples were fixed in formalin and were paraffin-embedded, and separate samples were frozen in liquid nitrogen and stored in the AP-HM tumor bank until use. Glioblastomas were histologically diagnosed according to the 2007 World Health Organization classification [38], and histological review of frozen samples by a pathologist (DFB) confirmed that all samples were located in the core of the tumor lesion and contained only tumor tissue, although necrosis might account for up to 30% of samples. IDH1 R132H protein expressions were routinely analyzed using immunohistochemistry and IDH1 R132H positive tumors were excluded. Genomic DNA was systematically extracted from frozen samples and *MGMT* promoter methylation and *EGFR* amplification status were evaluated as previously described (Figure 5) [39, 40].

### Tumor location

**Figure 6:**
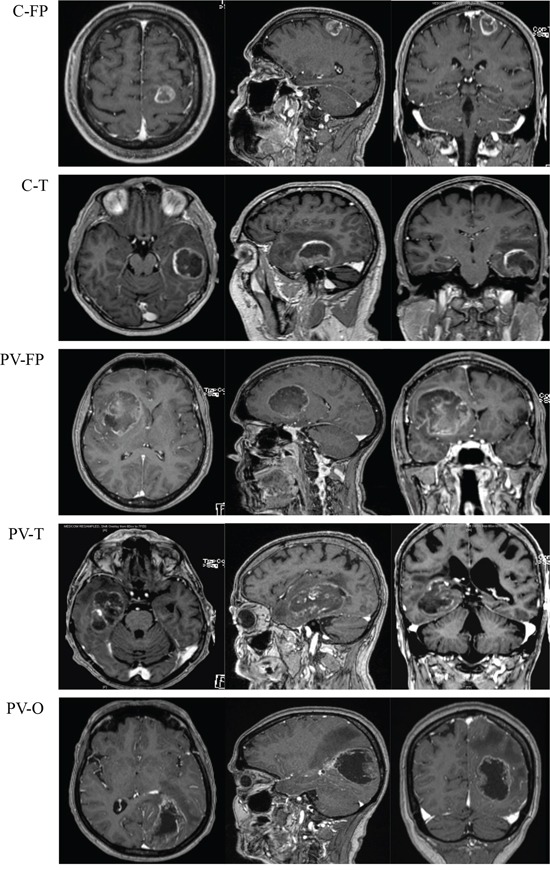
Magnetic resonance imaging with T1 enhancement (axial (left), sagittal (middle) and coronal (right)) of five tumor locations Abbreviations: Cortical fronto-parietal (C-FP), Cortical temporal (C-T), Periventricular fronto-parietal (PV-FP), Periventricular temporal (PV-T), Periventricular occipital (PV-O).

Preoperative MRI was performed on at least three axes (sagittal, axial, and coronal) using T1 without gadolinium injection, T1 with gadolinium injection, and T2 or FLAIR sequences. MRI data were reviewed by three physicians (DFB, ET, PM) and tumors were classified into cortical (C) (*n* = 24) and periventricular (PV) (*n* = 35) locations. All periventricular tumors presented with T1 enhancement in contact with lateral ventricles. Tumors were then divided according to locations and predominant lobar involvements of fronto-parietal (FP), temporal (T), or occipital (O) regions. Cortical tumors included C-FP (*n* = 19) and C-T (*n* = 5), and no cortical-occipital tumors were identified in this series. Finally, periventricular tumors were divided into fronto-parietal location (PV-FP, *n* = 12), temporal horn location (PV-T, *n* = 12), and posterior occipital horn location (PV-O, *n* = 11) based on the cerebral lobe involvement and ventricular anatomy (Figure 5 and Figure 6).

### RNA extraction

Total RNA was extracted from frozen tissues using TRI Reagent (Sigma-Aldrich, Paris, France) according to the manufacturer's instructions, and an improved version of the single-step total RNA isolation reagent developed by Chomczynski and Sacchi [41]. RNA samples were analyzed using a spectrophotometer (Nanodrop ND-1000 and Agilent 2100 bioanalyzer; Agilent Technologies, Massy, France). RNA samples with no evidence of ribosomal peak degradation and RIN values of 6–10 were used for real-time quantitative PCR analyses [42] after treatment with 1U ribonuclease-free deoxyribonuclease (Roche Applied Science, Meylan, France) at 37°C for 15 min.

### Real-time quantitative PCR (RT-qPCR)

RNA samples were processed using a LightCycler 480 instrument (Roche Applied Science) and a LightCycler 480 SYBR Green I Master Mix (Roche Applied Science). Briefly, total DNA-free RNA (1 μg) was reverse-transcribed into cDNA using 1 μg of random hexamers and Superscript II reverse transcriptase as recommended by the manufacturer (Invitrogen Life Technologies, Cergy Pontoise, France). Measurements were performed in triplicate for each sample, and relative expression ratios of target gene transcripts (*VEGFC*, *FLT4*, *MET*, *HGF*, *CHI3L1*, *PROM1*, *NOTCH1*, *DLL3*, *PDGFRA*, and *BCAN*) and reference gene transcripts (*18S*, *GAPDH*, and *ACTB*) were calculated using qPCR efficiencies and cycle threshold (Ct) deviations of tumor and normal adult brain samples (control: Agilent Technologies) [43]. RNA expression levels in glioblastoma samples were subsequently expressed as percentages compared to normal adult human brain samples corresponding to 100% of expression. Forward and reverse primers used for each gene are listed in Supplementary Table S4.

### Immunohistochemical analysis

Immunohistochemystry was performed on tissue microarrays (TMA) that were constructed from routinely processed formalin-fixed paraffin-embedded tumor material. Areas of viable and representative tumor following review of all blocks were marked by a pathologist (DFB) before inclusion in the TMA (3 × 0.6 mm cores for each tumor). TMA slides were simultaneously immunostained using Ventana Ultraview™ peroxydase system combined with the following primary antibody: anti-NOTCH1 (Abcam ab52627) [44]. NOTCH1 staining was scored negative or positive by two independent pathologists (DFB, DM).

### Statistical analysis

Qualitative variables are presented as frequencies and percentages, and continuous variables are presented as means (with standard errors), medians, and ranges. Correlations between tumor locations and RNA expression profiles or protein expression were analyzed using non-parametric Kruskal-Wallis and Mann-Whitney tests. The Chi-square test (or Fisher's exact test) was used to compare qualitative variables. Correlations between expression levels of molecular markers were evaluated using Spearman correlation coefficients. All reported *p*-values were two-sided, and differences were considered significant when *p* < 0.05. All statistical analyses were performed using PASW Statistics version 22.0 (IBM SPSS Inc., Chicago, IL, USA).

## SUPPLEMENTARY TABLES


