# Supplementary material for: Molecular heterogeneity of glioblastomas: does location matter?
Source: Oncotarget. 2015 Nov 30;7(1):902–13. doi: 10.18632/oncotarget.6433 (PMC4808041; doi:10.18632/oncotarget.6433)
Supplement: Supplementary file 1 [file oncotarget-07-0902-s001.pdf]

SUPPLEMENTARY TABLES

Supplementary Table S1: Mean RNA expression according to the five locations and for the entire population

Markers		Expression level					
		C-FP	C-T	PV-FP	PV-T	PV-O	Global
VEGFC	Mean	48,9	31,5	118,3	176,3	50,6	95,5
	SEM	9,82	4,9	34,4	44,7	12,1	11,2
FLT4	Mean	355,11	442,8	429,6	411,9	252,6	409,7
	SEM	61,3	95,9	67,1	70,2	29,3	26,7
MET	Mean	18,4	13,2	23,6	19,5	419,3	69,3
	SEM	5,6	3,7	4,1	4,9	292,9	37,1
HGF	Mean	191,5	190,2	359,2	349,9	268,9	313,8
	SEM	43,9	154,9	104,1	57,9	60,9	45,2
CHI3L1	Mean	7965,2	4146	14855,3	2541,4	9397,9	8204,3
	SEM	1645,2	586,4	4490,1	651,6	4094	1095,9
PROM1	Mean	103,5	132,9	81,4	219,3	145,8	139
	SEM	24,9	34,8	30,2	55,5	34,1	16,1
NOTCH1	Mean	316,8	207,5	240,9	435,5	310,6	313,1
	SEM	75,2	41,4	39,9	70,7	61,1	23,8
DLL3	Mean	264,9	135,8	357,8	390,7	232,7	365,1
	SEM	69,8	41,7	154,8	113,1	71,4	56,6
PDGFRA	Mean	507,4	258,2	3311,4	2433,2	1762,8	1209,9
	SEM	216,2	95,7	2195,8	1797,9	1024,9	404,5
BCAN	Mean	660,4	498,8	656,1	617,5	534,2	708,7
	SEM	103,9	121,7	221,7	158,8	140,2	68,7

Data are expressed as mean with Standard Error of the Mean (SEM).
Abbreviations: Cortical fronto-parietal (C-FP), Cortical temporal (C-T), Periventricular fronto-parietal (PV-FP), Periventricular temporal (PV-T), Periventricular occipital (PV-O)

Supplementary Table S2: RNA expression according to the periventricular tumor locations (Mann-Withney test)

	VEGFC		*FLT4*		*PROM1*		*NOTCH1*		*CHI3L1*	
	PV-FP	PV-O	PV-FP	PV-O	PV-FP	PV-O	PV-FP	PV-O	PV-FP	PV-O
PV-T	0,198	**0,004**	0,755	0,104	**0,033**	0,695	**0,028**	0,19	**0,006**	0,169
PV-O	**0,032**	-	**0,037**	-	0,059	-	0,566	-	0,235	-

P-values are presented. Bold entries indicate significant results.
Abbreviations: Periventricular fronto-parietal (PV-FP), Periventricular temporal (PV-T), Periventricular occipital (PV-O)

Supplementary Table S3: Correlation of RNA expression (Spearman test)

	MET	HGF	NOTCH1	FLT4	PDGFRA	BCAN	VEGFC	PROM1	CHI3L1	
SCC	-,330	,141	,487	,289	,353	,639	-,166	,654	-,543	**DLL3**
P-values	0,011		<0,001	0,026	0,006	<0,001		<0,001	<0,001	
SCC		,324	-,172	-,101	-,136	-,360	,296	-,313	,395	**MET**
P-values		0,012				0,005	0,023	0,016	0,002	
SCC			,275	,122	-,001	,015	,473	,147	-,041	**HGF**
P-values			0,035				<0,001			
SCC				,421	,276	,562	,121	,477	-,384	**NOTCH1**
P-values				0,001	0,035	<0,001		<0,001	0,003	
SCC					,032	,297	,422	,179	-,027	**FLT4**
P-values						0,022	0,001			
SCC						,207	-,092	,292	-,482	**PDGFRA**
P-values								0,025	<0,001	
SCC							-,186	,463	-,401	**BCAN**
P-values								<0,001	0,002	
SCC								-,194	,174	**VEGFC**
P-values										
SCC									-,535	**PROM1**
P-values									<0,001	

Significant spearman correlation coefficients (SCC) are underlined. *P*-values are below the data.

Supplementary Table S4: Sequences of primers used in RT-qPCR

Name	Sequence	Size/bp
18S	F: 5'-CTACCACATCCAAGGAAGGCA-3'<br>R: 5'-TTTTTCGTCACTACCTCCCCG-3'	71
GAPDH	F: 5'-CAAATTCCATGGCACCGTC-3'<br>R: 5'-CCCACTTGATTTTGGAGGGA-3'	101
ACTB	F: 5'-CCACACTGTGCCCATCTACG-3'<br>R: 5'-AGGATCTTCATGAGGTAGTCAGTCAG-3'	99
VEGFC	F: 5'-GATGCCTGGCTCAGGAAGA-3'<br>R: 5'-TGTCATGGAATCCATCTCTGTTGA-3'	72
FLT4	F: 5'-GGGCTTCTCCAGACCAAGA-3'<br>R: 5'-ACAAGATCTTCCATGGTCAGC-3'	44
MET	F: 5'-AAATGTGCATGAAGCAGGAA-3'<br>R: 5'-TCTCTGAATTAGAGCGATGTT-3'	62
HGF	F: 5'-TGATCCAAACATCCGAGTTG-3'<br>R: 5'-GCCATTCCCACGATAACAAT-3'	85
CHI3L1	F: 5'-GGGACCCTTGCCTACTATGA-3'<br>R: 5'-CGAGGATTCTATGGACTGTGG-3'	64
PROM1	F: 5'-GGGAGAACAATAATAGGATATTTTGAA-3'<br>R: 5'-CGATGCCACTTTCTCACTGAT-3'	75
NOTCH1	F: 5'-CGGGGCTAACAAAGATATGC-3'<br>R: 5'-CACCTTGGCGGTCTCGTA-3'	88
DLL3	F: 5'-CAACTGTGAGAAGAGGGTGGA-3'<br>R: 5'-CCAGGTCCAGGCAGAGTC-3'	71
PDGFRA	F: 5'-TGGTTGAAGGAACAGCCTATGG-3'<br>R: TGGCCGTGGGTTTTAGCAT-3'	81
BCAN	QUANTITECT (Ref: QT00088550)	144

Forward (F)/Reverse (R) primers and size of corresponding amplified fragment for each gene are listed. PCR conditions were as follows: 10 min at 95°C, followed by 35 cycles of 15 s at 95°C, 30 s at 67°C for 18S or 45 cycles of 15 s at 95°C, 30 s at 65°C for *GAPDH*, *ACTB*, *VEGFC* and *FLT4* or 20 s at 60°C for *MET*, *HGF* and *NOTCH1* or 30 s at 60°C for *CHI3L1*, *PROM1*, *PDGFRA* and *BCAN* or 20 s at 67°C for *DLL3*.
